# Intimate partner violence among pregnant women in Kenya: forms, perpetrators and associations

**DOI:** 10.1186/s12905-022-01761-7

**Published:** 2022-06-07

**Authors:** Mariella Stiller, Till Bärnighausen, Michael Lowery Wilson

**Affiliations:** Heidelberg Institute of Global Health (HIGH), University of Heidelberg, Im Neuenheimer Feld 130.3, 69120 Heidelberg, Germany

**Keywords:** Women’s health, Intimate partner violence, Pregnancy, Kenya, Associations, Demographic and health survey

## Abstract

**Background:**

Intimate Partner violence (IPV) among pregnant women is a significant problem of public health importance. Nevertheless, there are relatively few studies which have examined the phenomenon in sub-Saharan settings. The aim of this study was to provide an overview of the prevalence, perpetrators, and associated factors of IPV during pregnancy in Kenya.

**Methods:**

We were making use of the 2014 Kenyan Demographic and Health Survey (KDHS) data and included women and girls of reproductive age (15–49 years) who have ever been pregnant ($n=4331$). A weighted sample of respondents who have experienced violence during pregnancy ($n=397$) were selected for further bivariate and multivariable logistic regression analyses in order to examine the association between IPV and socio-demographic factors.

**Results:**

The prevalence of violence among pregnant women in Kenya was 9.2%, perpetrated mostly by the current husband or partner (47.6%), followed by the former husband or partner (31.5%). Physical violence was the most common (78.6%), followed by emotional (67.8%) and sexual (34.8%). Having one or two children ($\hbox {aOR} =0.68$; $\hbox {CI} = 0.53{-}0.88$), having secondary or higher education ($\hbox {aOR} = 0.53$; $\hbox {CI} = 0.40{-}0.69$) and being 18 years and above at first cohabitation ($\hbox {aOR} = 0.75$; $\hbox {CI}= 0.60{-}0.94$) and at sexual debut ($\hbox {aOR} = 0.65$; $\hbox {CI}=0.53{-}0.80$) were significantly associated with fewer reports of violence during pregnancy. Pregnant women who were divorced, separated or widowed ($\hbox {aOR} = 1.91$; $\hbox {CI} =1.47{-}2.47$), who were employed ($\hbox {aOR} = 1.34$; $\hbox {CI} =1.06{-}1.70$), who had witnessed their fathers beat their mothers ($\hbox {aOR} = 1.59$; $\hbox {CI} = 1.28{-}1.97$) and who had primary education ($\hbox {aOR} = 1.53$; $\hbox {CI} = 1.11{-}2.14$) were significantly more likely to experience violence.

**Conclusions:**

To prevent violence among pregnant women in Kenya, training health care providers should go hand in hand with interventions sensitising and mobilising community members, both addressing the socio-demographic drivers of IPV during pregnancy and directing a particular attention to the most vulnerable ones.

## Background

Intimate partner violence (IPV) has been acknowledged internationally as a persistent human rights violation and an urgent global health issue, which almost one in three women has experienced during their lifetime [1, 2]. Although violence against women is perpetrated by different actors, there is evidence that the main perpetrators are intimate male partners [2, 3]. IPV, also referred to as domestic violence or spouse abuse, can be defined as acts of physical aggression, sexual coercion, psychological/emotional abuse or controlling behaviours by a current or former partner or spouse [2].

Violence during pregnancy has been aligned with numerous adverse health outcomes, including not only physical injuries, sexual, reproductive, and mental health problems, but also maternal and infant death, homicide, and suicide [4–6]. In particular, the experience of IPV during pregnancy is significantly associated with increased child mortality and morbidity, preterm birth, low birth weight, miscarriage, stillbirth, unsafe abortion, sexually transmitted diseases (including HIV/AIDS), as well as unhealthy behaviour during pregnancy such as smoking, alcohol and drug abuse, and delayed prenatal care [4, 6–8]. Moreover, the vast majority of maternal deaths related to pregnancy complications, as well as neonatal and child deaths, occur in low- and middle-income countries [9, 10], where, in turn, the prevalence of IPV is highest worldwide [11–13].

These adverse health outcomes emphasise that pregnant women and their unborn children are particularly vulnerable to violence. Additionally, pregnancy can increase women’s economic and marital dependency on their partners, while exacerbating their vulnerability in abusive situations [14, 15]. Furthermore, pregnancy can involve increased stress levels due to increasing physical, psychological, and socio-economic burdens, which may also lead to decreased healthy mechanisms for coping with stress [16, 17]. These pregnancy-related stressors can put pregnant women at a higher risk for experiencing physical and verbal aggression in an intimate relationship [16, 17].

Globally, the prevalence of IPV during pregnancy was found to be ranging mostly from 4% in Thailand to 12% in provincial Tanzania and Bangladesh, with the lowest rate of 1% in high-income countries, such as Japan, and the highest rate of 28% in low-income countries, such as Peru [11]. In addition, a study of 19 countries worldwide reported a higher prevalence of IPV among pregnant women in Africa and Latin America with the highest rate of 13.5% in sub-Saharan Africa [12]. In Africa, almost two out of three women in profoundly affected areas have experienced violence by an intimate partner during their lifetime [18]. In a systematic review of thirteen African studies on IPV against pregnant women, it was reported that its overall prevalence was 15% with a range from 2 to 57% with the lowest rate in Nigeria and the highest in Uganda [13]. Similarly, an East African study indicated a prevalence of IPV against women of 14–39% with the highest percentage in Uganda [19].

In Kenya, the prevalence of IPV during pregnancy ranges from 34 to 67% across different health facilities, which is the highest among the available data from Africa [20–22]. Some health facility-based studies in Kenya revealed that 27–56% of women reported experiencing physical violence, between 10 and 30% were exposed to psychological violence, and 12–39% were subjected to sexual violence [20–22].

Kenya represents an important window into a specific African context, as there is relatively few literature on violence trends during pregnancy, particularly in areas where its prevalence among the general population is high [3, 13, 15]. In the broader Kenyan community, IPV is accepted and socialised as a normal and timeless tradition, thus, its practice is legitimised [15, 20, 23]. Moreover, most studies about IPV among pregnant women in Kenya were conducted in healthcare facilities [3, 20–22, 24], whereas research with population-based and nationally representative data is scarce. This can be particularly problematic due to the fact that pregnant women who are not able to access healthcare are often underrepresented in IPV studies [20]. Additionally, research has evidenced that women subjected to violence are less likely to use antenatal or delivery care in Kenya [25]. Further barriers for the detection of IPV in healthcare settings include the healthcare providers’ lack of screening skills, their negative attitudes toward violence survivors, their lack of information on IPV, time and staff [26–28].

Experiencing IPV during pregnancy is associated with several risk and preventive factors. Having a partner with tertiary education and being in a voluntary relationship were reported as protective factors against IPV [20]. Risk factors for experiencing IPV include woman’s low level of education, woman’s low socioeconomic status, young age (under 20 years), diagnosis of HIV, sexual risk factors such as having a partner with multiple concurrent sexual partners, history of violence such as experiencing violence during childhood, and alcohol abuse, both by women and partners [13]. Other associations with an increased risk for experiencing violence are having been witness to physical and sexual violence, being multiparous, dominance in decision-making of the male partner and infidelity by the women [3, 20]. However, different and sometimes even controversial findings were reported in the literature; therefore, this study aimed to assess the associated factors of IPV during pregnancy.

Emerging research suggests that there is a significant need for evidence-based interventions aimed at preventing IPV during pregnancy in Kenya, as well as raise awareness around this matter [11, 17, 18]. The urgency of this topic is also clearly apparent from being linked with the United Nations’ Sustainable Development Goals (SDGs). By 2030, SDG number 3 aims to ensure child and maternal health and reproductive health-care services, and SDG number 5 appeals for gender equality and women’s empowerment with the elimination of all forms of violence against women and girls [29]. To address these essential needs, it is necessary to provide a holistic analysis of IPV among pregnant Kenyan women.

## Methods

### Aim

The aim of the present study was to examine the prevalence, perpetrators, patterns and associated factors for IPV among pregnant women in Kenya.

### Setting

This study made use of data which was collected in the Republic of Kenya. The equatorial East African country is situated next to the Indian Ocean and shares borders with Tanzania, Uganda, South Sudan, Ethiopia and Somalia. Kenya has a population of approximately 52.5 million inhabitants of whom almost half a million are refugees and asylum seekers whose origin is mainly Somalia, South Sudan, the Democratic Republic of the Congo and Ethiopia [30]. With a GDP per capita of about 1800 US$, Kenya is classified as a lower-middle-income country [31]. Kenya’s mortality rate for children under 5 years old is 5.2% and the maternal mortality rate is 0.36% [32].

### Sample

This study utilises previously collected data from the Kenyan Demographic and Health Survey (DHS). The Kenyan DHS provides nationally representative information on Kenyan households, considering the social distribution of its neighbourhood residential stability, socioeconomic status, and diversity [33]. Data was collected in 2014 through the DHS-7 standardised woman’s and household questionnaires at the household level in different Kenyan households, implemented by the International Classification of Functioning, Disability and Health (ICF) and its partners [34]. Within the DHS sample, only one woman per household was randomly selected to participate in the Domestic Violence module in which she was interviewed about violence perpetrated by her current or most recent husband or partner.

For this research, the data of women and girls of reproductive age (15–49 years) who have ever been pregnant were included in the present study ($n = 4331$). From this sample, only respondents who reported having experienced violence during pregnancy ($n=397$) were selected for further bivariate and multivariable analyses.

Ethical procedures taken at the time of the data collection are based on the ethical and safety recommendations for research on domestic violence against women published by the World Health Organisation [35]. All methods were performed in accordance with the relevant guidelines and regulations. Further, the data used in this study are publicly available in anonymised form (www.dhsprogram.com/methodology/survey/survey-display-451.cfm). Additional information about the DHS program, questionnaires, procedures and methods can be obtained from the official website (www.dhsprogram.com).

### Measurements

The data used to inform this study was derived from the Woman’s Questionnaire and the DHS Domestic Violence module within the Household Questionnaire. In this study, the variable categories of interest included: 1) violence during pregnancy, 2) perpetrator characteristics, 3) different forms of violence, and 4) demographic background characteristics.

1. Violence during pregnancy, the dependent variable, was derived from the Domestic Violence module, in which women aged 15–49 years who have ever been pregnant were asked: *“Has anyone ever hit, slapped, kicked, or done anything else to hurt you physically while you were pregnant?”* with a yes-or-no-answer.
2. Furthermore, the same sample of women was asked the following question about the perpetrator/s: *“Who has done any of these things to physically hurt you while you were pregnant? Anyone else?”*. The possible answers were *“current husband/partner”, “mother/step-mother”, “father/step-father”, “sister/brother”, “daughter/son”, “other relative”, “former husband/partner”, “current boyfriend”, “former boyfriend”, “mother-in-law”, “father-in-law”, “other in-law”, “teacher”, “employer/someone at work”, “police/soldier”* and *“other”*. The possible answers “current husband/partner”, “former husband/partner”, “current boyfriend”, or “former boyfriend” were considered as intimate partners. All respondents who reported having experienced violence during pregnancy, regardless of the perpetrator, were selected for further analyses.
3. Because intimate partners are the main perpetrators of violence against women [3], the DHS survey focuses especially on them. Spousal violence by partners for currently married women and by one’s most recent partner for formerly married women was detected by asking all ever-married women the following yes-or-no-questions: When the woman answered *“yes”*, she was asked the follow-up question *“How often did this happen during the last 12 months: often, only sometimes, or not at all?”*. To distinguish the different forms of violence, items a to c typify emotional/psychological violence, items d to j physical violence, and items k to m sexual violence, as described in Table 1. If a woman answered one or more of the items of a violence form with the answer “yes”, the evidence of this type of violence is demonstrated [32]. Women could select multiple items, hence these different forms of violence are not mutually exclusive.
4. Socio-demographic background characteristics were measured in association with the experience of violence during pregnancy in general and for each type of violence specifically, whether psychological, physical or sexual one. These results were analysed and interpreted to ascertain the risk and preventive factors for IPV among pregnant women. Basic socio-demographic factors included age, age at first cohabitation, age at sexual debut, religion, residence, region, marital status, number of living children, employment, education and wealth quintile, whether a woman is afraid of her partner, timing of first violence after marriage, and whether a woman’s father “beat” her mother; all these factors were asked in the DHS household questionnaire. In addition, the husbands’ characteristics were included in this analysis, such as the partner’s age, his education, occupation, and alcohol consumption.

**Table 1:** Dependent variable derivation from the DHS survey data (2014)

	Did your (last) husband/partner ever:	Form of violence
(a)	Say or do something to humiliate you in front of others?	Emotional
(b)	Threaten to hurt or harm you or someone you care about?	Violence
(c)	Insult you or make you feel bad about yourself?	
(d)	Push you, shake you, or throw something at you?	
(e)	Slap you?	
(f)	Twist your arm or pull your hair?	Physical
(g)	Punch you with his fist or with something that could hurt you?	Violence
(h)	Kick you, drag you, or beat you up?	
(i)	Try to choke you or burn you on purpose?	
(j)	Threaten or attack you with a knife, gun, or other weapon?	
(k)	Physically force you to have sexual intercourse with him when you did not want to?	Sexual
(l)	Physically force you to perform any other sexual acts you did not want to?	Violence
(m)	Force you with threats or in any other way to perform sexual acts you did not want to?	

### Statistical analyses

We examined the prevalence of violence during pregnancy, the distribution of perpetrators and of the three different forms of violence (physical, emotional and sexual) by lifetime and in the 12 months preceding the survey (often or sometimes). We analysed the distribution of selected background characteristics within the dependent variables (violence during pregnancy and physical, emotional and sexual violence). These independent variables include age, age at first cohabitation, age at sexual debut, religion, residence, region, marital status, number of living children, employment, education and wealth quintile, whether a woman is afraid of her partner, timing of first violence after marriage, whether a woman’s father “beat” her mother, the partner’s age, his education, occupation, and alcohol consumption. For the measures of bivariate associations between the socio-demographic characteristics and violence, we used the chi-square for categorical variables and the *t*-test for continuous variable (age). The results of the bivariate analyses were reported as proportions.

Only the background variables found to be statistically significantly associated in the bivariate analyses were used for the multivariable logistic regression modeling. Multivariable regression models were conducted to explore the association between women’s socio-demographic background characteristics and their experience of violence during pregnancy (model 1). In complementary, we conducted three additional regression models to assess women’s background characteristics associated with physical IPV (model 2), emotional IPV (model 3), and sexual IPV (model 4). The models present the measures of association as odds ratios (ORs) with their corresponding 95% confidence intervals (CI). Statistical significance both for the preliminary and the confirmatory analyses was considered at a *p*-value of $<0.05$ as well as $<0.005$. ORs were adjusted for women’s age, education, ethnicity, employment and place of residence (urban/rural). We used the R Statistical Environment version 3.6.1 for the analyses [36].

## Results

A total of 5657 women aged 15–49 years participated in the Domestic Violence module of the Kenyan Demographic and Health Survey 2014 of whom 4331 women reported ever being pregnant. The prevalence of violence among pregnant women in the present study was 9.2% ($n= 397$).

### Perpetrators

Table 2 shows the total percent distribution of reported perpetrators of violence among pregnant women. Among pregnant women, the most commonly reported perpetrator of violence was the current husband or partner (47.6%, $n=189$), followed by the former husband or partner (31.5%, $n=125$). Taken together, violence during pregnancy was with 80.2% perpetrated by intimate partners (current and former husband/partner or boyfriend). Furthermore, persons who committed violence among pregnant women were relatives (7.6%, $n=30$) other than parents, siblings and children, followed by in-laws (3.5%, $n=14$) other than the woman’s parents-in-law, mother or step-mother (2.8%, $n=11$), sister or brother (2.3%, $n=9$) and teacher (1%, $n=4$).

**Table 2:** Distribution of reported perpetrators of violence among pregnant women in Kenya (2014)

Perpetrator	Number of women	Prevalence (in %)
Current husband/partner	189	47.6
Mother/step-mother	11	2.8
Father/step-father	2	0.5
Sister/brother	9	2.3
Daughter/son	1	0.3
Other relative	30	7.6
Former husband/partner	125	31.5
Current boyfriend	1	0.3
Former boyfriend	3	0.8
Mother in-law	2	0.5
Father in-law	1	0.3
Other in-law	14	3.5
Teacher	4	1.0
Employer/someone at work	0	0.0
Police/soldier	1	0.3
Other	15	3.8
Women reporting violence during pregnancy	397	100.0

Women can report more than one person who committed the violence

### Different forms of violence

As Table 3 describes, almost 4 out of 5 pregnant women (78.6%) experienced any physical violence, more than two thirds (67.8%) any emotional violence, and more than one third (34.8%) any sexual violence. The major form of violence was committed often or sometimes through slapping (45.4%), pushing, shaking or throwing something (36.2%), and kicking, dragging or beating up (33.0%). Even more than 1 in 10 pregnant women who experienced physical violence were often or sometimes threatened or attacked with a knife, gun or other weapon (13.8%), and were tried to be choked or burned on purpose (10.9%). Insulting or making feel bad often or sometimes (42.9%) was the main form of emotional violence pregnant women suffered, followed by saying or doing something to humiliate her in front of others (32.7%). The majority of pregnant women experienced sexual violence often or sometimes as they were physically forced to have sexual intercourse when they did not want to (23.5%).

**Table 3:** Distribution of different forms of violence during pregnancy among women in Kenya (2014), weighted

Type of violence	Ever	Often	Sometimes	Often or sometimes
*Emotional violence*				
Any emotional violence	67.8			
Said or did something to humiliate her in front of others		14.8	17.9	32.7
Threatened to hurt or harm her or someone she cared about		12.1	16.2	28.2
Insulted her or made her feel bad about her		20.6	22.2	42.9
*Sexual violence*				
Any sexual violence	34.8			
Physically forced her to have sexual intercourse with him when she did not want to		10.3	13.3	23.5
Physically forced her to perform any other sexual acts she did not want to		4.7	6.8	11.5
Forced her with threats or in any other way to perform sexual acts she did not want to		5.4	4.3	9.7
*Physical violence*				
Any physical violence	78.6			
Pushed her, shook her, or threw something at her		14.7	21.4	36.2
Slapped her		20.3	25.1	45.4
Twisted her arm or pulled her hair		10.9	13.1	24.0
Punched her with his fist or with something that could hurt her		13.8	16.8	30.6
Kicked her, dragged her, or beat her up		16.6	16.3	33.0
Tried to choke her or burn her on purpose		4.5	6.4	10.9
Threatened her or attacked her with a knife, gun, or other weapon		4.8	9.0	13.8
Any form of physical and, or sexual violence	80.0			
Any form of emotional and, or physical and, or sexual violence	85.9			
Total 15–49 years	397			

All variables are expressed as proportions (in %). Women could select multiple items, hence these different forms of violence are not mutually exclusive

### Bivariate analysis

Table 4 presents the percentage distribution of socio-demographic characteristics of women who did and did not experience violence during pregnancy. The mean age of those women was 32.1 years (SD 8.0 years), and almost a half (45.8%) were aged 25–34 years. At first cohabitation, the majority of pregnant IPV survivors was aged 18 years or above (58.2%), or 15–17 years (28.3%). At sexual debut, most respondents were 18 years old or older (47.0%), or aged 15–17 years (27.1%). 4 in 5 women (75.3%) were Protestants or other Christians than Roman Catholics and more than 1 in 2 lived in rural areas (57.6%) and in the Western region (55.5%) which includes the former Kenyan provinces Nyanza, Rift Valley and Western. 61.3% of women who experienced violence during pregnancy were married or lived together with the partner, and 41.3% had 1 or 2 children. Most of the participants were employed (77.4%), had a primary education (65.2%) and were either from the poorer or poorest (40.4%) or the richer or richest (41.8%) wealth quintiles. More than half of IPV survivors were sometimes or most of the time afraid (61.0%) of their partner, 42.0% had witnessed their fathers abusing their mothers, and more than a quarter (26.4%) experienced violence for the first time in the first 2 years after marriage. The majority of intimate partners were 35 years old or older (35.8%), had a primary (49.1%) or secondary or higher education (43.1%), had a manual or agricultural or household and domestic work (72.8%), and often drank alcohol (35.9%). We found very similar distributions of socio-demographic characteristics of Kenyan women subjected to emotional, sexual and physical violence, as presented in Tables 5, 6 and 7.

**Table 4:** Distribution of selected variables according to violence during pregnancy among women in Kenya (2014), weighted

Variable	Violence during pregnancy (*n* = 397)	No violence during pregnancy (*n* = 3914)	*p*-value	Chi-square or *t*-value
Age (mean)	32.1 (8.0)	31.5 (8.0)	0.158	1.413
*Age in 5-year-group*				
15–19	2.3	4.0	0.103	10.551
20–24	17.4	17.0		
25–29	29.0	24.2		
30–34	16.8	18.4		
35–39	14.0	15.5		
40–44	9.7	12.1		
45–49	10.7	8.7		
*Age at first cohabitation*				
$\le$ 14	9.4	7.6	< **0**.**001**	− 4.666
15–17	28.3	23.9		
18+	58.2	58.9		
*Age at sexual debut*				
$\le$ 14	18.4	13.3	0.080	-1.754
15–17	27.1	24.3		
18+	47.0	53.4		
*Religion*				
Roman Catholic	18.8	20.2	0.084	7.948
Protestant, other Christian	75.3	70.1		
Muslim	3.8	6.5		
No religion	2.1	2.8		
Other religion	0.1	0.4		
*Residence*				
Urban	42.4	39.9	0.366	0.816
Rural	57.6	60.1		
*Region*				
Central	5.7	13.7	< **0**.**001**	105.050
Eastern	17.3	27.8		
Western	55.5	48.7		
Nairobi	21.6	9.8		
*Marital status*				
Married or living together	61.3	77.2	< **0**.**001**	200.750
Divorced, separated, widowed	18.6	13.2		
*Number of living children*				
0	1.5	3.3	**0**.**004**	3.128
1–2	41.3	46.3		
3–4	33.8	28.9		
5+	23.4	21.6		
Employment (yes)	77.4	71.6	**0**.**015**	5.910
*Education*				
No education	6.2	8.8	< **0**.**001**	18.490
Primary	65.2	55.1		
Secondary or higher	28.7	36.1		
*Wealth quintile*				
Poorer or poorest	40.4	35.4	< **0**.**001**	24.135
Middle	17.8	19.5		
Richer or richest	41.8	45.1		
*Woman afraid of partner*				
Never	34.9	60.1	< **0**.**001**	195.320
Sometimes	29.7	20.9		
Most of the time	31.3	9.3		
*Timing of first violence after marriage*				
0 year	16.1	4.3	< **0**.**001**	4.047
1–2 years	26.4	10.4		
3–4 years	16.7	6.5		
5–10 years	14.7	8.0		
11+ years	5.3	2.2		
Woman’s father beat mother	42.0	37.1	0.163	3.631
*Partner’s age*				
15–24	1.5	2.3	0.075	1.787
25–34	22.9	27.6		
35+	35.8	46.8		
*Partner’s education*				
No education	5.4	6.8	**0**.**027**	9.815
Primary	49.1	42.1		
Secondary or higher	43.1	44.7		
*Partner working*				
Did not work	1.6	1.4	**0**.**010**	18.401
Professional, technical, managerial or clerical or services	25.5	24.4		
Manual or agricultural or household and domestic	72.8	74.0		
*Partner’s alcohol consumption*				
Never	0.0	0.3	< **0**.**001**	91.103
Often	35.9	10.3		
Sometimes	18.0	21.0		
Total 15–49 years	391	3914		

All variables are expressed as proportions (in %) except for age (mean and standard deviation). Age variables are presented in years

Chi-square or *t*-value and *p*-value concern the violence during pregnancy

Bold indicates *p*-value < 0.05 is statistically significant

**Table 5:** Distribution of selected variables according to emotional violence among women in Kenya (2014), weighted

Variable	Emotional violence (*n* = 1301)	No emotional violence (*n* = 2717)	No violence in any form (*n* = 2008)	*p*-value	Chi-square or *t*-value
Age (mean)	32.7 (8.0)	31.2 (7.9)	31.3 (7.8)	< **0**.**001**	− 5.644
*Age in 5-year-group*					
15–19	1.6	3.3	2.8	**0**.**001**	21.804
20–24	13.7	17.2	17.1		
25–29	25.2	24.8	24.3		
30–34	19.2	18.8	19.1		
35–39	16.9	15.2	15.6		
40–44	13.2	12.0	12.6		
45–49	10.3	8.6	8.5		
*Age at first cohabitation*					
$\le$ 14	9.8	7.7	7.7	< **0**.**001**	3.836
15–17	28.9	25.5	24.5		
18+	61.4	66.8	67.8		
*Age at sexual debut*					
$\le$ 14	17.1	10.9	10.4	< **0**.**001**	8.916
15–17	33.7	31.8	29.2		
18+	49.1	57.3	60.4		
*Religion*					
Roman Catholic	18.7	19.9	19.4	< **0**.**001**	65.066
Protestant, other Christian	75.8	68.5	68.1		
Muslim	2.4	8.8	9.6		
No religion	2.4	2.5	2.6		
Other religion	0.7	0.2	0.3		
*Residence*					
Urban	39.4	39.5	39.7	0.998	< **0**.**001**
Rural	60.6	60.5	60.3		
*Region*					
Central	11.0	13.8	14.3	< **0**.**001**	134.55
Eastern	22.2	30.6	31.5		
Western	53.8	46.6	46.2		
Nairobi	13.0	9.0	8.0		
*Marital status*					
Married or living together	74.1	87.7	89.2	< **0**.**001**	192.630
Divorced, separated, widowed	25.9	12.3	10.8		
*Number of living children*					
0	3.4	5.8	3.2	< **0**.**001**	4.494
1–2	36.8	43.4	46.2		
3–4	34.7	28.8	29.0		
5+	25.1	22.0	21.5		
Employment (yes)	80.2	68.6	66.8	< **0**.**001**	59.001
*Education*					
No education	6.5	10.7	10.9	< **0**.**001**	29.693
Primary	61.3	54.1	52.0		
Secondary or Higher	32.2	35.2	37.1		
*Wealth quintile*					
Poorer or Poorest	38.0	36.4	35.3	< **0**.**001**	35.834
Middle	21.5	17.7	17.8		
Richer or Richest	40.5	45.9	46.9		
*Woman afraid of partner*					
Never	42.2	74.4	80.3	< **0**.**001**	456.280
Sometimes	32.8	19.2	15.5		
Most of the time	24.8	6.3	4.2		
*Timing of first violence after marriage*					
0 year	11.0	3.5	0.0	0.178	35.861
1–2 years	25.2	6.9	0.0		
3–4 years	16.2	4.1	0.0		
5–10 years	17.2	5.6	0.0		
11+ years	5.4	1.3	0.0		
Woman’s father beat mother	45.3	33.8	30.5	< **0**.**001**	49.548
*Partner’s age*					
15–24	2.2	3.2	2.8	< **0**.**001**	− 4.417
25–34	23.3	33.6	32.9		
35+	47.6	50.6	53.1		
*Partner’s education*					
No education	4.9	8.7	8.3	< **0**.**001**	22.457
Primary	50.2	45.1	43.4		
Secondary or Higher	43.8	45.3	46.9		
*Partner working*					
Did not work	1.8	1.4	1.6	< **0**.**001**	34.689
Professional, technical, managerial or clerical or services	23.7	25.4	27.1		
Manual or agricultural or household and domestic	74.5	73.1	70.2		
*Partner’s alcohol consumption*					
Never	0.4	0.3	0.3	< **0**.**001**	105.400
Often	25.8	7.9	5.7		
Sometimes	23.5	22.3	20.4		
Total 15–49 years	1301	2717	2008		

All variables are expressed as proportions (in %) except for age (mean and standard deviation). Age variables are presented in years

Chi-square or *t*-value and *p*-value concern the emotional violence

Bold indicates *p*-value < 0.05 is statistically significant

**Table 6:** Distribution of selected variables according to sexual violence among women in Kenya (2014), weighted

Variable	Sexual violence (*n* = 534)	No sexual violence (*n* = 3484)	No violence in any form (*n*=2008)	*p*-value	Chi-square or *t*-value
Age (mean)	32.3 (7.7)	31.6 (8.0)	31.3 (7.8)	0.058	− 1.900
*Age in 5-year-group*					
15–19	1.4	3.0	2.8	0.199	8.580
20–24	14.8	16.3	17.1		
25–29	26.8	24.6	24.3		
30–34	17.8	19.1	19.1		
35–39	18.1	15.4	15.6		
40–44	12.3	12.4	12.6		
45–49	8.9	9.2	8.5		
*Age at first cohabitation*					
$\le$ 14	10.2	8.1	7.7	< **0**.**001**	3.628
15–17	29.1	26.2	24.5		
18+	60.7	65.7	67.8		
*Age at sexual debut*					
$\le$ 14	18.0	12.1	10.4	< **0**.**001**	5.644
15–17	38.7	31.5	29.2		
18+	43.0	56.4	60.4		
*Religion*					
Roman Catholic	19.6	19.5	19.4	< **0**.**001**	21.212
Protestant, other Christian	76.1	70.1	68.1		
Muslim	2.5	7.4	9.6		
No religion	1.7	2.6	2.6		
Other religion	0.2	0.4	0.3		
*Residence*					
Urban	41.8	39.1	39.7	0.253	1.308
Rural	58.2	60.9	60.3		
*Region*					
Central	7.7	13.7	14.3	< **0**.**001**	127.970
Eastern	20.3	29.0	31.5		
Western	55.6	47.9	46.2		
Nairobi	16.4	9.3	8.0		
*Marital status*					
Married or living together	72.0	85.0	89.2	< **0**.**001**	70.560
Divorced, separated, widowed	28.0	15.0	10.8		
*Number of living children*					
0	2.9	5.3	3.2	0.002	3.503
1–2	38.4	41.6	46.2		
3–4	33.8	30.3	29.0		
5+	24.9	22.7	21.5		
Employment (yes)	79.3	71.3	66.8	< **0**.**001**	14.105
*Education*					
No education	6.5	9.8	10.9	< **0**.**001**	33.949
Primary	67.3	54.8	52.0		
Secondary or higher	43.9	35.5	37.1		
*Wealth quintile*					
Poorer or Poorest	38.0	36.8	35.3	0.063	8.930
Middle	22.7	18.3	17.8		
Richer or Richest	39.3	44.9	46.9		
*Woman afraid of partner*					
Never	29.3	69.3	80.3	< **0**.**001**	416.400
Sometimes	35.4	21.8	15.5		
Most of the time	35.3	8.8	4.2		
*Timing of first violence after marriage*					
0 year	20.7	3.7	0.0	< **0**.**001**	4.564
1–2 years	30.5	10.1	0.0		
3–4 years	18.5	6.4	0.0		
5–10 years	22.7	7.3	0.0		
11+ years	6.0	2.1	0.0		
Woman’s father beat mother	53.3	35.1	30.5	< **0**.**001**	66.422
*Partner’s age*					
15–24	1.0	3.2	2.8	0.028	− 2.198
25–34	25.8	30.9	32.9		
35+	44.7	50.4	53.1		
*Partner’s education*					
No education	4.8	7.8	8.3	< **0**.**001**	24.970
Primary	51.2	46.1	43.4		
Secondary or Higher	43.5	44.9	46.9		
*Partner working*					
Did not work	1.2	1.6	1.6	< **0**.**001**	33.153
Professional, technical, managerial or clerical or services	23.7	24.8	27.1		
Manual or agricultural or household and domestic	74.0	72.7	70.2		
*Partner’s alcohol consumption*					
Never	0.1	0.3	0.3	< **0**.**001**	56.716
Often	30.7	11.1	5.7		
Sometimes	23.6	22.6	20.4		
Total 15–49 years	534	3484	2008		

All variables are expressed as proportions (in %) except for age (mean and standard deviation). Age variables are presented in years

Chi-square or *t*-value and *p*-value concern the sexual violence

Bold indicates *p*-value < 0.05 is statistically significant

**Table 7:** Distribution of selected variables according to physical violence among women in Kenya (2014), weighted

Variable	Physical violence (*n* = 1431)	No physical violence (*n* = 2586)	No violence in any form (*n* = 2008)	*p*-value	Chi-square or *t*-value
Age (mean)	32.6 (8.0)	31.2 (7.9)	31.3 (7.8)	< **0**.**001**	− 5.530
*Age in 5-year-group*					
15–19	1.3	3.6	2.8	< **0**.**001**	31.106
20–24	14.5	17.0	17.1		
25–29	24.4	25.1	24.3		
30–34	19.2	18.8	19.1		
35–39	17.1	15.0	15.6		
40–44	12.7	12.3	12.6		
45–49	10.8	8.3	8.5		
*Age at first cohabitation*					
$\le$ 14	10.2	7.4	7.7	< **0**.**001**	7.579
15–17	29.8	24.8	24.5		
18+	60.0	67.8	67.8		
*Age at sexual debut*					
$\le$ 14	18.2	10.0	10.4	< **0**.**001**	8.213
15–17	36.3	30.3	29.2		
18+	45.4	59.7	60.4		
*Religion*					
Roman Catholic	20.8	18.8	19.4	< **0**.**001**	45.002
Protestant, other Christian	73.2	69.6	68.1		
Muslim	3.3	8.7	9.6		
No religion	2.5	2.5	2.6		
Other religion	0.2	0.5	0.3		
*Residence*					
Urban	41.8	39.1	39.7	< **0**.**001**	17.224
Rural	58.2	60.9	60.3		
*Region*					
Central	10.8	14.0	14.3	< **0**.**001**	127.970
Eastern	24.2	29.9	31.5		
Western	53.0	46.7	46.2		
Nairobi	12.0	9.3	8.0		
*Marital status*					
Married or living together	75.3	87.7	89.2	< **0**.**001**	126.660
Divorced, separated, widowed	24.7	12.3	10.8		
*Number of living children*					
0	2.0	6.7	3.2	< **0**.**001**	4.605
1–2	35.4	44.5	46.2		
3–4	36.3	27.7	29.0		
5+	26.3	21.2	21.5		
Employment (yes)	80.1	68.1	66.8	< **0**.**001**	65.947
*Education*					
No education	8.2	9.9	10.9	< **0**.**001**	79.035
Primary	64.4	52.0	52.0		
Secondary or Higher	27.3	38.1	37.1		
*Wealth quintile*					
Poorer or Poorest	41.4	34.5	35.3	< **0**.**001**	60.375
Middle	21.0	17.8	17.8		
Richer or Richest	37.6	47.7	46.9		
*Woman afraid of partner*					
Never	38.9	77.8	80.3	< **0**.**001**	692.870
Sometimes	34.2	17.8	15.5		
Most of the time	26.6	4.4	4.2		
*Timing of first violence after marriage*					
0 year	13.3	1.9	0.0	< **0**.**001**	3.545
1–2 years	33.4	1.4	0.0		
3–4 years	21.0	0.7	0.0		
5–10 years	23.7	1.5	0.0		
11+ years	8.2	0.3	0.0		
Woman’s father beat mother	46.9	32.3	30.5	< **0**.**001**	91.986
*Partner’s age*					
15–24	1.5	3.6	2.8	< **0**.**001**	− 3.709
25–34	26.2	32.5	32.9		
35+	46.8	51.2	53.1		
*Partner’s education*					
No education	7.0	7.6	8.3	< **0**.**001**	68.742
Primary	53.1	43.3	43.4		
Secondary or Higher	39.1	47.9	46.9		
*Partner working*					
Did not work	1.5	1.6	1.6	< **0**.**001**	62.188
Professional, technical, managerial or clerical or services	19.7	27.3	27.1		
Manual or agricultural or household and domestic	78.2	69.9	70.2		
*Partner’s alcohol consumption*					
Never	0.4	0.3	0.3	< **0**.**001**	101.73
Often	26.2	6.8	5.7		
Sometimes	26.0	20.9	20.4		
Total 15–49 years	1431	2586	2008		

All variables are expressed as proportions (in %) except for age (mean and standard deviation). Age variables are presented in years

Chi-square or *t*-value and *p*-value concern the physical violence

Bold indicates *p*-value < 0.05 is statistically significant

### Multivariable analysis

Table 8 presents the logistic regression analysis of experiencing violence during pregnancy. Tables 9, 10 and 11 show the multivariable analyses of emotional, sexual and physical violence. There is evidence that living in rural areas [11, 37] and being employed [38, 39] entails a higher risk for women to experience IPV. Likewise, Kenyan Luo and Luhya women were significantly more likely to report having experienced IPV than women of other ethnicities [40]. Therefore, the models of multivariable analysis were adjusted for woman’s age, woman’s education, woman’s ethnicity, woman’s employment and woman’s place of residence (urban, rural). We found statistically significant associations for all selected variables, with the exception of woman’s and partner’s age, residence and partner’s working.

**Table 8:** Multivariable analysis of violence among pregnant women in Kenya (2014), adjusted and weighted

Variable	aOR	95% CI	*p*-value
Age (continuous)	1.01	0.99–1.02	0.321
*Age in 5-year-group*			
15–19$^{\mathrm{r}}$			
20–24	1.90	0.95–4.37	0.095
25–29	1.90	0.96–4.31	0.090
30–34	1.63	0.81–3.75	0.205
35–39	1.73	0.86–3.99	0.157
40–44	2.03	0.99–4.73	0.073
45–49	2.02	0.97–4.77	0.080
*Age at first cohabitation*			
$$\le 14^{\mathrm{r}}$$			
15–17	1.27	1.01–1.58	0.042*
18+	0.75	0.60–0.94	0.011*
*Age at sexual debut*			
$$\le 14^{\mathrm{r}}$$			
15–17	1.21	0.97–1.50	0.096
18+	0.65	0.53–0.80	< 0.001**
*Religion*			
Roman Catholic$^{\mathrm{r}}$			
Protestant, other Christian	1.13	0.87–1.48	0.353
Muslim	0.44	0.27–0.68	< 0.001**
No religion	1.03	0.51–1.90	0.934
Other religion	1.09	0.17–4.01	0.914
*Residence*			
Urban$^{\mathrm{r}}$			
Rural	0.95	0.76–1.19	0.679
*Region*			
Central$^{\mathrm{r}}$			
Eastern	0.47	0.37–0.61	< 0.001**
Western	1.95	1.57–2.44	< 0.001**
Nairobi	3.51	1.85–6.53	< 0.001**
*Marital status*			
Married or living together$^{\mathrm{r}}$			
Divorced, separated, widowed	1.91	1.47–2.47	< 0.001**
*Number of living children*			
0$^{\mathrm{r}}$			
1–2	0.68	0.53–0.88	0.003**
3–4	1.23	0.99–1.52	0.058
5	1.28	0.97–1.67	0.075
Employment (Yes)	1.34	1.06–1.70	0.016*
*Education*			
No education$^{\mathrm{r}}$			
Primary	1.53	1.11–2.14	0.011*
Secondary or higher	0.53	0.40–0.69	< 0.001**
*Wealth quintile*			
Poorer or poorest$^{\mathrm{r}}$			
Middle	1.00	0.71–1.39	0.989
Richer or Richest	0.75	0.57–0.98	0.035*
*Woman afraid of partner*			
Never$^{\mathrm{r}}$			
Sometimes	3.38	2.61–4.38	< 0.001**
Most of the time	6.38	4.85–8.41	< 0.001**
Woman’s father beat mother	1.59	1.28–1.97	< 0.001**
*Partner’s age*			
15–24$^{\mathrm{r}}$			
25–34	1.12	0.82–1.52	0.485
35+	0.90	0.64–1.25	0.519
*Partner’s education*			
No education$^{\mathrm{r}}$			
Primary	2.14	1.47–3.18	< 0.001**
Secondary or higher	0.85	0.66–1.09	0.196
*Partner working*			
Did not work$^{\mathrm{r}}$			
Professional, technical, managerial or clerical or services	0.79	0.59–1.04	0.099
Manual or agricultural or household and domestic	1.28	0.98–1.69	0.071
*Partner’s alcohol consumption*			
Never$^{\mathrm{r}}$			
Often	4.04	2.97–5.54	< 0.001**
Sometimes	0.26	0.19–0.36	< 0.001**

Model is adjusted for woman’s age, education, ethnicity, employment and place of residence (urban, rural). Age variable is presented in years

aOR, adjusted Odds Ratio. 95% CI, 95% Confidence Interval. $^{\mathrm{r}}$ Reference group

*p-value $$<0.05$$. **p-value $$<0.005$$

**Table 9:** Multivariate analysis of emotional violence among pregnant women in Kenya (2014), adjusted and weighted

Variable	aOR	95% CI	*p*-value
Age (continuous)	1.02	1.01–1.02	< 0.001**
*Age in 5-year-group*			
15–19$^{\mathrm{r}}$			
20–24	1.42	0.92–2.23	0.122
25–29	1.42	0.94–2.22	0.110
30–34	1.61	1.06–2.53	0.032*
35–39	1.71	1.12–2.70	0.016*
40–44	1.77	1.14–2.82	0.013*
45–49	1.87	1.19–3.00	0.008*
*Age at first cohabitation*			
$\le$ 14$^{\mathrm{r}}$			
15–17	1.26	1.09–1.46	0.002**
18+	0.72	0.63–0.83	< 0.001**
*Age at sexual debut*			
$\le$ 14$^{\mathrm{r}}$			
15–17	1.31	1.14–1.51	< 0.001**
18+	0.62	0.54–0.70	< 0.001**
*Religion*			
Roman Catholic$^{\mathrm{r}}$			
Protestant, other Christian	1.08	0.91–1.27	0.381
Muslim	0.28	0.21–0.38	< 0.001**
No religion	0.89	0.57–1.36	0.594
Other religion	2.41	0.90–6.59	0.079
*Residence*			
Urban$^{\mathrm{r}}$			
Rural	1.02	0.88–1.17	0.827
*Region*			
Central$^{\mathrm{r}}$			
Eastern	0.60	0.52–0.69	< 0.001**
Western	1.68	1.47–1.92	< 0.001**
Nairobi	1.83	1.18–2.82	0.006*
*Marital status*			
Married or living together$^{\mathrm{r}}$			
Divorced, separated, widowed	1.97	1.66–2.35	< 0.001**
*Number of living children*			
$$0^{\mathrm{r}}$$			
1–2	0.81	0.70–0.95	0.008*
3–4	1.26	1.10–1.44	< 0.001**
5	1.06	0.89–1.26	0.507
Employment (yes)	1.99	1.71–2.32	< 0.001**
*Education*			
No education$^{\mathrm{r}}$			
Primary	1.56	1.27–1.92	< 0.001**
Secondary or higher	0.81	0.69–0.93	< 0.005**
*Wealth quintile*			
Poorer or Poorest$^{\mathrm{r}}$			
Middle	1.12	0.91–1.38	0.283
Richer or Richest	0.65	0.55–0.76	< 0.001**
*Woman afraid of partner*			
Never$^{\mathrm{r}}$			
Sometimes	3.27	2.80–3.83	< 0.001**
Most of the time	7.30	5.99–8.92	< 0.001**
Woman’s father beat mother	1.82	1.59–2.08	< 0.001**
*Partner’s age*			
15–24$^{\mathrm{r}}$			
25–34	0.86	0.72–1.04	0.118
35+	1.16	0.96–1.42	0.132
*Partner’s education*			
No education$^{\mathrm{r}}$			
Primary	1.94	1.52–2.47	< 0.001**
Secondary or higher	0.88	0.76–1.03	0.108
*Partner working*			
Did not work$^{\mathrm{r}}$			
Professional, technical, managerial or clerical or services	0.77	0.65–0.90	0.002**
Manual or agricultural or household and domestic	1.24	1.06–1.45	0.009*
*Partner’s alcohol consumption*			
Never$^{\mathrm{r}}$			
Often	2.77	2.23–3.45	< 0.001**
Sometimes	0.38	0.30–0.47	< 0.001**

Model is adjusted for woman’s age, education, ethnicity, employment and place of residence (urban, rural). Age variable is presented in years

aOR, adjusted Odds Ratio. 95% CI, 95% Confidence Interval. $^{\mathrm{r}}$Reference group. *p-value $<0.05$. **p-value $<0.005$

**Table 10:** Multivariate analysis of sexual violence among pregnant women in Kenya (2014), adjusted and weighted

Variable	aOR	95% CI	*p*-value
Age (continuous)	1.00	0.99–1.02	0.406
*Age in 5-year-group*			
15–19$^{\mathrm{r}}$			
20–24	1.60	0.84–3.36	0.181
25–29	1.72	0.92–3.57	0.112
30–34	1.57	0.83–3.29	0.193
35–39	1.92	1.02–4.04	0.060
40–44	1.71	0.88–3.64	0.135
45–49	1.60	0.81–3.45	0.201
*Age at first cohabitation*			
$\le$ 14$^{\mathrm{r}}$			
15–17	1.29	1.06–1.58	0.012*
18+	0.71	0.58–0.86	< 0.001**
*Age at sexual debut*			
$\le$ 14$^{\mathrm{r}}$			
15–17	1.34	1.10–1.62	0.003**
18+	0.58	0.48–0.70	< 0.001**
*Religion*			
Roman Catholic$^{\mathrm{r}}$			
Protestant, other Christian	1.09	0.87–1.38	0.466
Muslim	0.29	0.18–0.45	< 0.001**
No religion	0.83	0.43–1.49	0.547
Other religion	1.40	0.31–4.45	0.608
*Residence*			
Urban$^{\mathrm{r}}$			
Rural	0.90	0.74–1.10	0.311
*Region*			
Central$^{\mathrm{r}}$			
Eastern	0.60	0.48–0.74	< 0.001**
Western	1.67	1.38–2.03	< 0.001**
Nairobi	2.60	1.50–4.42	< 0.001**
*Marital status*			
Married or living together$^{\mathrm{r}}$			
Divorced, separated, widowed	1.87	1.48–2.33	< 0.001**
*Number of living children*			
$$0^{\mathrm{r}}$$			
1–2	0.97	0.78–1.21	0.801
3–4	1.14	0.94–1.38	0.173
5	0.98	0.77–1.25	0.858
Employment (yes)	1.84	1.48–2.31	< 0.001**
*Education*			
No education$^{\mathrm{r}}$			
Primary	1.85	1.36–2.55	< 0.001**
Secondary or higher	0.67	0.54–0.84	< 0.001**
*Wealth quintile*			
Poorer or Poorest$^{\mathrm{r}}$			
Middle	1.17	0.88–1.56	0.277
Richer or Richest	0.63	0.50–0.80	< 0.001**
*Woman afraid of partner*			
Never$^{\mathrm{r}}$			
Sometimes	3.65	2.90–4.59	< 0.001**
Most of the time	8.56	6.71–10.94	< 0.001**
Woman’s father beat mother	2.05	1.69–2.49	< 0.001**
*Partner’s age*			
15–24$^{\mathrm{r}}$			
25–34	1.08	0.82–1.41	0.589
35+	1.12	0.84–1.49	0.449
*Partner’s education*			
No education$^{\mathrm{r}}$			
Primary	2.52	1.75–3.72	< 0.001**
Secondary or higher	0.89	0.72–1.10	0.293
*Partner working*			
Did not work$^{\mathrm{r}}$			
Professional, technical, managerial or clerical or services	0.85	0.66–1.07	0.166
Manual or agricultural or household and domestic	1.18	0.94–1.48	0.167
*Partner’s alcohol consumption*			
Never$^{\mathrm{r}}$			
Often	3.01	2.30–3.94	< 0.001**
Sometimes	0.35	0.26–0.45	< 0.001**

Model is adjusted for woman’s age, education, ethnicity, employment and place of residence (urban, rural). Age variable is presented in years

aOR, adjusted Odds Ratio. 95% CI, 95% Confidence Interval. $^{\mathrm{r}}$Reference group. *p-value $<0.05$. **p-value $<0.005$

**Table 11:** Multivariate analysis of physical violence among pregnant women in Kenya (2014), adjusted and weighted

Variable	aOR	95% CI	*p*-value
Age (continuous)	1.01	1.01–1.02	< 0.001**
*Age in 5-year-group*			
15–19$^{\mathrm{r}}$			
20–24	2.09	1.36–3.34	0.001**
25–29	2.21	1.45–3.48	< 0.001**
30–34	2.36	1.53–3.74	< 0.001**
35–39	2.19	1.42–3.49	< 0.001**
40–44	2.50	1.60–4.02	< 0.001**
45–49	2.74	1.74–4.44	< 0.001**
*Age at first cohabitation*			
$\le$ 14$^{\mathrm{r}}$			
15–17	1.27	1.10–1.45	< 0.001**
18+	0.73	0.64–0.83	< 0.001**
*Age at sexual debut*			
$\le$ 14$^{\mathrm{r}}$			
15–17	1.35	1.18–1.54	< 0.001**
18+	0.56	0.49–0.64	< 0.001**
*Religion*			
Roman Catholic$^{\mathrm{r}}$			
Protestant, other Christian	0.99	0.84–1.16	0.861
Muslim	0.36	0.28–0.47	< 0.001**
No religion	0.69	0.45–1.05	0.089
Other religion	0.77	0.26–2.11	0.625
*Residence*			
Urban$^{\mathrm{r}}$			
Rural	1.13	0.99–1.30	0.071
*Region*			
Central$^{\mathrm{r}}$			
Eastern	0.66	0.57–0.76	< 0.001**
Western	1.41	1.24–1.60	< 0.001**
Nairobi	2.78	1.81–4.27	< 0.001**
*Marital status*			
Married or living together$^{\mathrm{r}}$			
Divorced, separated, widowed	1.97	1.65–2.34	< 0.001**
*Number of living children*			
$$0^{\mathrm{r}}$$			
1–2	0.86	0.74–1.00	0.043*
3–4	1.28	1.12–1.45	< 0.001**
5	1.05	0.89–1.24	0.534
Employment (yes)	1.85	1.60–2.14	< 0.001**
*Education*			
No education$^{\mathrm{r}}$			
Primary	1.24	1.03–1.50	0.022*
Secondary or higher	0.59	0.51–0.68	< 0.001**
*Wealth quintile*			
Poorer or Poorest$^{\mathrm{r}}$			
Middle	1.13	0.92–1.38	0.239
Richer or Richest	0.66	0.56–0.78	< 0.001**
*Woman afraid of partner*			
Never$^{\mathrm{r}}$			
Sometimes	3.82	3.28–4.45	< 0.001**
Most of the time	10.30	8.38–12.71	< 0.001**
Woman’s father beat mother	2.03	1.78–2.32	< 0.001**
*Partner’s age*			
15–24$^{\mathrm{r}}$			
25–34	1.21	1.02–1.45	0.032*
35+	0.87	0.72–1.05	0.157
*Partner’s education*			
No education$^{\mathrm{r}}$			
Primary	1.39	1.12–1.72	0.003**
Secondary or higher	0.76	0.66–0.89	< 0.001**
*Partner working*			
Did not work$^{\mathrm{r}}$			
Professional, technical, managerial or clerical or services	0.71	0.60–0.84	< 0.001**
Manual or agricultural or household and domestic	1.40	1.20–1.64	< 0.001**
*Partner’s alcohol consumption*			
Never$^{\mathrm{r}}$			
Often	2.69	2.16–3.37	< 0.001**
Sometimes	0.39	0.31–0.49	< 0.001**

Model is adjusted for woman’s age, education, ethnicity, employment and place of residence (urban, rural). Age variable is presented in years

aOR, adjusted Odds Ratio. 95% CI, 95% Confidence Interval. $^{\mathrm{r}}$Reference group. *p-value $<0.05$. **p-value $<0.005$

Women aged 15–17 years at first cohabitation ($\hbox {aOR} = 1.27$; $\hbox {CI} =1.01{-}1.58$) were more likely to experience violence during pregnancy, compared to women aged 18 years and above at first cohabitation ($\hbox {aOR}=0.75$; $\hbox {CI} = 0.60{-}0.94$) who were less likely to experience violence during pregnancy. Respondents aged 18 years and above at sexual debut ($\hbox {aOR} = 0.65$; $\hbox {CI} = 0.53{-}0.80$) had a lower risk for experiencing violence when they were pregnant. Being Muslim ($\hbox {aOR} = 0.44$; $\hbox {CI}=0.27{-}0.68$) was associated with lower odds of experiencing violence. Women having one or two children ($\hbox {aOR}=0.68$; $\hbox {CI}=0.53{-}0.88$) were less likely to be subjected to IPV during pregnancy. Having secondary or higher education ($\hbox {aOR} = 0.53$; $\hbox {CI}=0.40{-}0.69$) and belonging to the richer or richest wealth quintile ($\hbox {aOR} = 0.75$; $\hbox {CI}=0.57{-}0.98$) were significantly associated with fewer reports of violence during pregnancy. Women living in the Western region were 1.95 times more likely ($\hbox {CI} =1.57{-}2.44$), and in Nairobi 3.51 times more likely ($\hbox {CI}=1.85{-}6.53$) to experience violence during pregnancy than those living in the Central region, while living in the Eastern region was protective ($\hbox {aOR}=0.47$; $\hbox {CI}=0.37{-}0.61$). Pregnant women who were divorced, separated, or widowed were more likely to experience violence ($\hbox {aOR}=1.91$; $\hbox {CI}=1.47{-}2.47$). Women’s employment was associated with more reports of violence during pregnancy ($\hbox {aOR}=1.34$; $\hbox {CI}=1.06{-}1.70$). Respondents who had witnessed their fathers beat their mothers were 1.59 times more likely ($\hbox {CI}=1.28{-}1.97$) to be subjected to IPV than those who had not witnessed paternal abuse. In addition, pregnant women who were sometimes or most of the time afraid of their partners had an increased risk for experiencing violence ($\hbox {aOR}=3.38$; $\hbox {CI}=2.61{-}4.38$ for women who were sometimes afraid, and $\hbox {aOR}=6.38$; $\hbox {CI}=4.85{-}8.41$ for those who were most of the time afraid). Primary education of both, women ($\hbox {aOR}=1.53$; $\hbox {CI}=1.11{-}2.14$) and their partners ($\hbox {aOR}=2.14$; $\hbox {CI}=1.47{-}3.18$), was associated with higher odds of violence during pregnancy.

## Discussion

The present study reports on the prevalence and perpetrators, as well as the different forms and associated factors of IPV among pregnant women in Kenya. This research found significant associations between the experience of violence during pregnancy in Kenya and various personal, partnership, sociocultural, and environmental factors. These findings are consistent with an ‘ecological framework’. This ‘ecological model’ understands violence against women as a result of multiple factors which interact at an individual, relationship, community, and society level [2, 41].

We revealed that almost one in ten women (9.2%) have experienced violence during pregnancy in Kenya. Although this rate is high, it is lower compared to prevalence rates of 28–67% reported in different health facility based studies in Kenya [3, 20–22, 24]. Clinic-based studies generally show a higher number of women reporting violence vis-à-vis population-based ones [3]. Women might be more favorable to disclose violence in healthcare settings due to ensured privacy and healthcare providers’ attentiveness and active enquiry of abuse [3, 42]. In the present research, respondents could also have deprived abuse due to their fear that participating in interviews could lead to further violence. Additionally, the cultural acceptance of violence within the broader Kenyan community [15] might contribute to women withholding to report the violence they experience. As a result, the violence rates found in this study could also be underestimated. Furthermore, Ethiopia’s Tigray conflict, which has destabilised the Horn of Africa [43, 44], may provoke negative social, economic, and political effects which could possibly exacerbate existing violence [45, 46] among pregnant women in Kenya and the surrounding region.

Our study indicates that violence during pregnancy was mostly (80.2%) perpetrated by intimate partners (current or former husband/partner or boyfriend) of which the current husband/partner was the most common perpetrator (47.6%). This is consistent with previous studies which evidence that IPV is the most common form of violence perpetrated against women [2, 3, 13, 47]. These findings might be linked to the strong patriarchal norms deeply rooted within some Kenyan communities [3, 15, 20]. In Kenya’s society (particularly in rural areas), a husband or partner can be legitimised to abuse his wife as a way of “disciplining” her [20]. As Mutisya et al. point out, “the belief in the social superiority of a man, man’s right to assert over a woman, and the belief that women should tolerate violence to save a relationship/marriage” are rooted in Kenya’s broader community [3]. These sociocultural beliefs are predictors of increased violence during pregnancy in Kenya [3].

Physical violence emerged as the most prevalent form of violence, which is consistent with other studies implemented in Kenya. However, we revealed a higher prevalence (78.6%) than in previous studies conducted in different Kenyan health facilities (in Kisumu and West Pokot County) ranging from 10 to 42% [3, 20, 21]. This high prevalence of physical violence is of concern, since this type of violence can have particularly detrimental impacts on the pregnancy, including maternal and neonatal death [7].

We ascertained that more than two thirds (67.8%) of pregnant women have experienced emotional violence - which is consistent with previous research findings in health facility based studies in Kenya (55.8%) [21]. Likewise, we found higher rates of psychological violence than in two Kenyan health facility based studies in Kisumu (29%) and Uasin Gishu County (27.4%) [20, 22]. This high rate of emotional violence is alarming, as this form of violence is associated with postnatal depression, anxiety, and other adverse mental health problems [6, 48] - which can limit a woman’s ability to care for herself and for her child [22]. Furthermore, psychological violence is very subtle and it can be hard for survivors to prove it [22].

The prevalence of sexual violence during pregnancy (34.8%) was similar to the rates disclosed in Kenyan health facilities in West Pokot County (39.2%) [21], and higher than in Kisumu (12%) and Uasin Gishu County (13%) [20, 22]. The divergent estimates could be attributed to methodological differences between studies, such as the study design, setting and the instruments used. Whereas we examined the different forms of violence among women who reported having ever experienced violence during pregnancy, other authors investigated prevalences within the previous year [3], in the current pregnancy [21] or of ever being exposed to any form of IPV [20], which might explain our higher rates.

Consistent with the existing literature of Kenya [20, 21, 49] and other African countries [47, 50], we found no significant associations between women’s age and the experience of violence during pregnancy in general. Furthermore, we revealed that young age (15–17 years) at first cohabitation was positively associated with all forms of IPV among pregnant women, which is also consistent with the existing literature [51], whereas an age of 18 years and above at first cohabitation was negatively associated with IPV. Similarly, women being 18 years and older at sexual debut were less likely to experience violence during pregnancy. A possible explanation of these patterns might be that violence tends to begin early after marriage (59.2% within the first 4 years, Table 4). This is consistent with a study which revealed that in sub-Saharan Africa, the median onset of spousal violence was 2 years after marriage [52]. Additionally, it must be taken into account that more than one third of women were minors (17 years or younger) when they lived together for the first time with their partner or had their sexual debut. Researchers expound that a younger age at first marriage/cohabitation or sexual debut might be related to less decision in choosing a husband, less time to gain a strong self-awareness outside the marriage, less educational attainment, and, therefore, more risk for being intimidated and abused by an intimate partner [53, 54]. In addition, younger women might have a lower social prestige than older women, and therefore might be less resilient to violence [11] and more likely to experience violent behavior [55], which, as aforementioned, could be aggravated by the vulnerability that pregnancy itself implies [15–17].

Being Muslim was significantly associated with fewer reports of violence during pregnancy, which is consistent with some existing studies in Kenya [22] and sub-Saharan Africa [56]. In addition, research in East-Africa [19], Nigeria [57] and the U.S. [58] confirm the significant association between religion and IPV among women. Although many religious groups reject violence, it could be that the strict norms within the Islamic religion might prohibit violence against women rigorously. Moreover, in our study, Muslims were underrepresented (3.8%, Table 4) and Christians were over-represented (94.1%), which is not exactly in line with the 2019 population and housing census in which 11% of Kenya’s population was Muslim and 86% Christian [59]. Therefore, these results should be interpreted cautiously. Nevertheless, there is few literature considering the correlation between religious affiliation and IPV among women in Kenya, and pregnant women in particular. Hence, it might be of interest for future researchers to explore this association as religion could be either a risk or a protective factor for IPV.

We found no association between place of residence (urban/rural) and IPV during pregnancy, which is in line with existing literature [20, 60]. In contrast, on one hand, other studies revealed that living in rural areas implies a higher risk for experiencing IPV [11, 37], whereas on the other hand, living in urban settings was found to be a risk factor for IPV as well [2, 51, 61]. Consistent with these findings, our study revealed that pregnant women living in Nairobi and the Western region were more likely to experience all forms of violence than in the Central region, whereas living in the Eastern region was a protective factor. A study conducted in South-Western Kenya [49] argues that the IPV prevalence there is higher than in overall Kenya, which could have multi-factorial reasons. Compared to overall Kenya, the Western region is characterised by higher poverty, poorer health outcomes, higher prevalence of HIV, lower education and higher polygamy rates, of which the two latter are reported as being associated with higher risk for experiencing IPV [20, 49, 62]. Additionally, Kenyan women of Luo and Luhya ethnicities, who are located in Western Kenya, were significantly more likely to be subjected to IPV than women of other ethnicities [40]. However, due to the paucity of substantial literature regarding the association between region, residence and IPV in Kenya, more research is needed to shed more light on this issue.

Being divorced, separated, or widowed was a significant risk factor for pregnant women for suffering violence, as evidenced by the literature [11]. Divorce, separation, and being pregnant while living without the partner might provoke social stigmatization, which might make women particularly vulnerable to violence during pregnancy. Additionally, Kenyan families are often unwilling to readopt their married daughters after divorce and to offer them protection back at home [49]. This lower social status in combination with limited social support and refuge might expose pregnant women living alone to an increased risk for experiencing violence [49]. As our data precludes causal inference, it could also be that divorced or widowed women might simply be more likely to report abuse due to their absence of fear against retaliation from their partners.

Having one or two children was found to be protective against IPV during pregnancy, whereas having three or four children was a predictor of emotional and physical IPV, which is consistent with a prior study [20]. Women having three or more children might be particularly dependent on their intimate partners. This economic dependence might aggravate particularly during pregnancy, as pregnant women often cannot work, especially during later stages of pregnancy. Moreover, marital dependence might exacerbate during pregnancy due to higher emotional and physical burdens, thus, pregnant women might rely particularly on their partners’ support for raising, providing, and caring for their children. These marital and economic dependencies might put pregnant women at higher risk for being and staying in abusive relationships [14].

Corresponding to previous findings in sub-Saharan Africa [19, 38, 39, 51, 56, 63], pregnant women who were employed were more likely to experience violence. This phenomenon arises in particular when employment differences between genders exist, more precisely women who are paid when their partner is not [38]. This might be explained by the theories of social exchange and relative resource: women with increased economic empowerment could challenge the men’s status and, therefore, be more likely to experience violence [14]. In turn, a man might abuse her partner to control her and uphold the dominant household status if he lacks economic empowerment [14]. By contrast, other researchers revealed that unemployed women were at higher risk for being abused by their partners, compared to their working peers [11, 64, 65]. This could be elucidated by the marital dependency theory which states that women with lower economic resources might be more dependent on a male partner and unable to change or end a relationship and, therefore, being exposed to an increased risk of violence [14].

Going hand in hand with the aforementioned women’s employment status, their education status plays an important role as well: Our research revealed that primary education of both, women and their partners, was significantly associated with more reports of violence during pregnancy, compared to those with no education. Consistently, some authors support this association [13, 14, 22, 66]. Furthermore, pregnant women having secondary or higher education had lower odds of violence. This effect is well-described in the existing literature [11, 14, 51, 62, 67] and may be explained by the increased access to different social and cultural norms in which partner violence is dismissed [63]. It might be also the case that higher educated women might have more decision-making in choosing a husband, might be more appreciated by their partners and might have a greater bargaining power within a partnership [53, 54]. In addition, higher educational attainment might increase women’s and men’s skills to resolve conflicts, and expand IPV survivors’ awareness about their rights and opportunities to reject abuse [53, 54, 63].

Belonging to the richer or richest wealth quintile was significantly negatively associated with violence during pregnancy, compared to the poorest or poorer wealth quintile, which is consistent with existing research [14, 61, 65]. These results are consistent with other studies which reported woman’s low wealth status as a risk factor for experiencing IPV in Kenya [51] and sub-Saharan Africa [13, 68, 69]. An elucidation of this phenomenon might be that a woman with a higher wealth status might be more financially independent from their partners and, therefore, she could more easily leave her abusive partner and be less likely to condone violence [14, 15]. Belonging to a richer wealth quintile could be particularly protective against violence, as pregnancy and the future child can entail a higher economic burden for pregnant women and their families [15]. Moreover, a higher wealth status might be often related to a higher educational attainment, which is also negatively associated with IPV; this might be especially the case in low- and middle-income countries such as Kenya, where higher education and universities are often private and cost-intensive [70]. However, in other sub-Saharan African countries, violence against women cuts across all wealth quintiles and, therefore, the ending of violence does not require a focus on wealth status alone but a holistic approach instead [69].

Having a partner working as a professional, technical, managerial, clerical or in services was negatively associated with emotional and physical violence during pregnancy, whereas having a partner who had a manual, agricultural or household and domestic work was positively associated. The latter professional fields may be connected to lower income and education than the first-mentioned. Hence, these results are comprehensible, as belonging to the poorest or poorer wealth quintile and primary education of both, women and their partners, were risk factors for violence among pregnant women, as aforementioned. Consistently, two health facility based studies in Kenya stated that women having an intimate partner with a stable or formal job had lower odds of violence than self-employed workers [3, 22].

In our study, pregnant women who were sometimes or most of the time afraid of their partners were more likely to be subjected to IPV, as evidenced by other researchers [50, 65]. Fear of the partner could be a precursor and, simultaneously, a consequence of violence during pregnancy [65]. The experience of violence could result in heightened fear, which, in turn, could intimidate pregnant women and deprive them of power, and consequently, might exacerbate the cycle of ongoing abuse [65].

Coinciding with previous studies [3, 11, 20, 22, 65], pregnant women who had witnessed their fathers abuse their mothers had an increased risk for experiencing violence. Pregnant women who had witnessed maternal abuse during childhood might have internalised such violence as normal and part of life or marriage [20]. The patriarchal shaped Kenyan society might contribute to this normalization of violence [1, 20]. Furthermore, women who had witnessed violence perpetrated by their fathers against their mothers might learn - consciously or unconsciously - behaviors and ways of thinking, which puts them at consequent risk for being abused later in life as well [66].

Having a partner aged between 25 and 34 years was a significant risk factor for physical violence among pregnant women as observed in a previous study in Kenya [20]. In addition, no association was found between partner’s age and emotional or sexual violence among pregnant women which is consistent with a Kenyan study [21]. Younger men might injure or threaten pregnant women in a more severe and frequent way due to their greater physical strength and health, compared to older men. Moreover, it could be the case that elder men are less violent because they might fear loneliness or might be dependent on their wives’ caregiving due to age-related illness or frailty. Men of older age might also have acquired more maturity and better conflict resolution skills within a marriage or partnership than men of younger age [3]. However, partner’s age is a controversially discussed factor regarding IPV. Whereas some researchers evidenced the association between partner’s age and IPV [19], other researchers do not [50].

The odds of violence were higher among pregnant women whose partners often drank alcohol, which is a commonly and globally observed association [3, 11, 13, 20, 21, 50, 62, 65]. In addition, we revealed lower odds of violence among pregnant women whose partners sometimes consumed alcohol. This association deviates from some existing literature [3, 62, 65]. There is evidence that partner’s excessive alcohol abuse contributes to a higher occurrence, severity and frequency of IPV [16, 71]. As pregnancy can be a challenging and stressful phase in life, some men might cope with pregnancy-related stressors by increasing their alcohol consumption, which, in turn, can lead to more perpetration of violence [72]. Nevertheless, attention must be paid to the missing distinction of the frequency of alcohol consumption between often and sometimes in many studies. Furthermore, the imprecise appraisal of alcohol intake may drive an association to the null, hence it makes it difficult to assess alcohol consumption by using questionnaires [73].

## Conclusion

Whereas previous IPV studies were mostly conducted in healthcare facilities in Kenya, the present study used nationally representative and globally comparable data, and contributes towards a deeper understanding of IPV during pregnancy in Kenya by adding insight into its prevalence, perpetrators and associated factors. The findings of this study might constitute a stronger basis for policy-makers to address the essential need for prevention of abuse during pregnancy in Kenya.

Consistent with an ’ecological framework’ [41], this research discovered significant associations between the experience of violence during pregnancy in Kenya and various individual, relationship, family, community, and society factors, which interact with each other. Practitioners are encouraged to take these socio-demographic drivers of IPV into account when creating a stronger advocacy and designing action against violence among pregnant women in Kenya. Reducing poverty, ensuring educational attainment, diminishing pregnant women’s marital and economic dependencies on their partners, and strengthening their decision-making capacity are recommended interventions to prevent IPV. Training health care providers might be conducive to ensure routine IPV screening within pre- and perinatal care [74]. This should go hand in hand with interventions sensitising and mobilising community members, equally engaging women and men, to foster the rejection and elimination of IPV during pregnancy in Kenya [75]. Researchers and practitioners should direct a particular attention to the most vulnerable ones, including pregnant women who are minors at first cohabitation and sexual debut, who are divorced, separated, or widowed, multiparous, employed, having a history of family violence, living in Nairobi and Western Kenya, and having a partner with excessive alcohol abuse.

These research findings must be interpreted in light of study design limitations, and the cross-sectional nature of the data limits the possibility of making causal conclusions. More research is needed to assess the association between IPV among pregnant women in Kenya and their religion, region and residence. To elucidate the complex social context of IPV, further research should be done on women’s lack of empowerment and male attitudes and beliefs that contribute to IPV among women during pregnancy in Kenya.

## Data Availability

This study utilises previously collected data from the Kenyan Demographic and Health Survey (DHS) 2014. The data used in this study are publicly available in anonymised form (www.dhsprogram.com/methodology/survey/survey-display-451.cfm). Additional information about the DHS program, questionnaires, procedures and methods can be obtained from the official website (www.dhsprogram.com).
